# Delayed ileal neobladder fistula caused by bladder stones: a case report

**DOI:** 10.1186/s12894-022-01040-5

**Published:** 2022-06-17

**Authors:** Dongming Lu, Yongyang Wu, Shangfan Liao, Xueping Xie, Dingjun Zhu, Shuchao Ye

**Affiliations:** 1 Department of Urology, Affiliated Sanming First Hospital, Fujian Medical University, Sanming, 365000 Fujian China; 2 Department of Urology, Sun Yat-Sen Memorial Hospital, Sun Yat-sen, Guangzhou, 510655 Guangdong China

**Keywords:** Ileal neobladder, Fistula, Radical cystectomy, Bladder stone, Case report

## Abstract

**Background:**

Ileal neobladder fistula is a rare complication after radical cystectomy, with an incidence of approximately 0.7%. At present, there are scattered reports of vesicoileal fistula, but there are no reports of ileal neobladder fistula (INF) caused by bladder stones. In this paper, a case of ileal neobladder fistula caused by chronic stimulation of bladder stones was successfully diagnosed and treated.

**Case presentation:**

A 68-year-old man who had undergone radical cystectomy and an orthotopic ileal neobladder procedure 10 years prior presented with refractory diarrhoea and oliguria and was diagnosed with ileal neobladder fistula caused by chronic stimulation of bladder stones. We performed fistulectomy, cystotomy, partial ileectomy, and end-to-end ileal anastomosis, and the patient recovered and was discharged after the operation.

**Conclusion:**

Urinary calculi are delayed complications of orthotopic neobladder construction after total cystectomy. Bladder stones are a rare complication of ileal neovesical fistula, which can cause neovesical cutaneous fistula. It is difficult to diagnose through routine examination and easily misdiagnosed as acute gastroenteritis. Surgery is an effective treatment for INF and can achieve a good prognosis.

## Background

Urinary fistula after radical cystectomy (RC) and orthotopic ileal neobladder is clinically rare. At present, there are scattered reports of vesicoileal fistula, but there are no reports of ileal neobladder fistula (INF) caused by bladder stones.

Ileal neobladder fistula is a rare complication after radical cystectomy, with an incidence of approximately 0.7% [1]. Its clinical manifestations are atypical and easily misdiagnosed. Relevant literature reports are mostly scattered case reports. The aetiology of INF is unclear. The early cause of INF may be local damage to the bowel, and the overlap of the ileal end-to-end anastomosis site with the neobladder suture of the ileum may be one of the causes of INF [2, 3]. In addition, local pelvic radiotherapy, long-term indwelling urinary catheters and local recurrence of tumours are possible causes of delayed INF [4].

Delayed INF due to ileal neobladder has not been reported. In this paper, a case of ileal neobladder fistula caused by chronic stimulation of bladder stones was successfully diagnosed and treated, and the report is as follows.

## Case presentation

The patient, a 68-year-old male, was admitted to our medical centre in November 2021 with 1 month of “draining watery stools”. More than 1 month prior, the patient had diarrhoea without obvious cause and passed yellow watery stools 7–8 times a day, more than 10 times a day. Half a month prior, the patient's diarrhoea did not improve and was accompanied by dull pain in the right lower quadrant. He went to a local hospital to consider “acute gastroenteritis or gastrointestinal dysfunction” and received “oral antibiotics” and other treatments, but the diarrhoea did not improve significantly. The patient developed oliguria 16 h prior and was referred to our hospital.

In 2011, due to high-grade urothelial invasive carcinoma of the bladder, he was treated in our centre and underwent "laparoscopic total cystectomy and “M” type ileal neobladder"; the catheter was removed 1 month after the operation. The voiding pattern was abdominal pressure voiding. During the 5-year follow-up period, the patient showed no local tumour recurrence by pelvic MRI or CT. After 2016, the patient did not visit the doctor for re-examination.

**Fig. 1:**
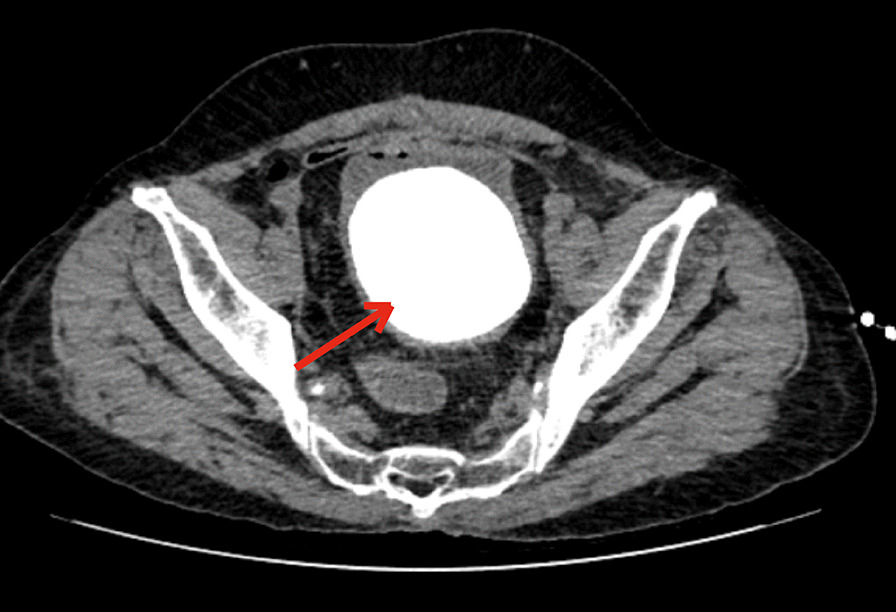
Nonenhanced pelvic CT scan. Notes: The red arrow shows a large stone (approximately 9.0 cm × 9.0 cm) in the neobladder

**Fig. 2:**
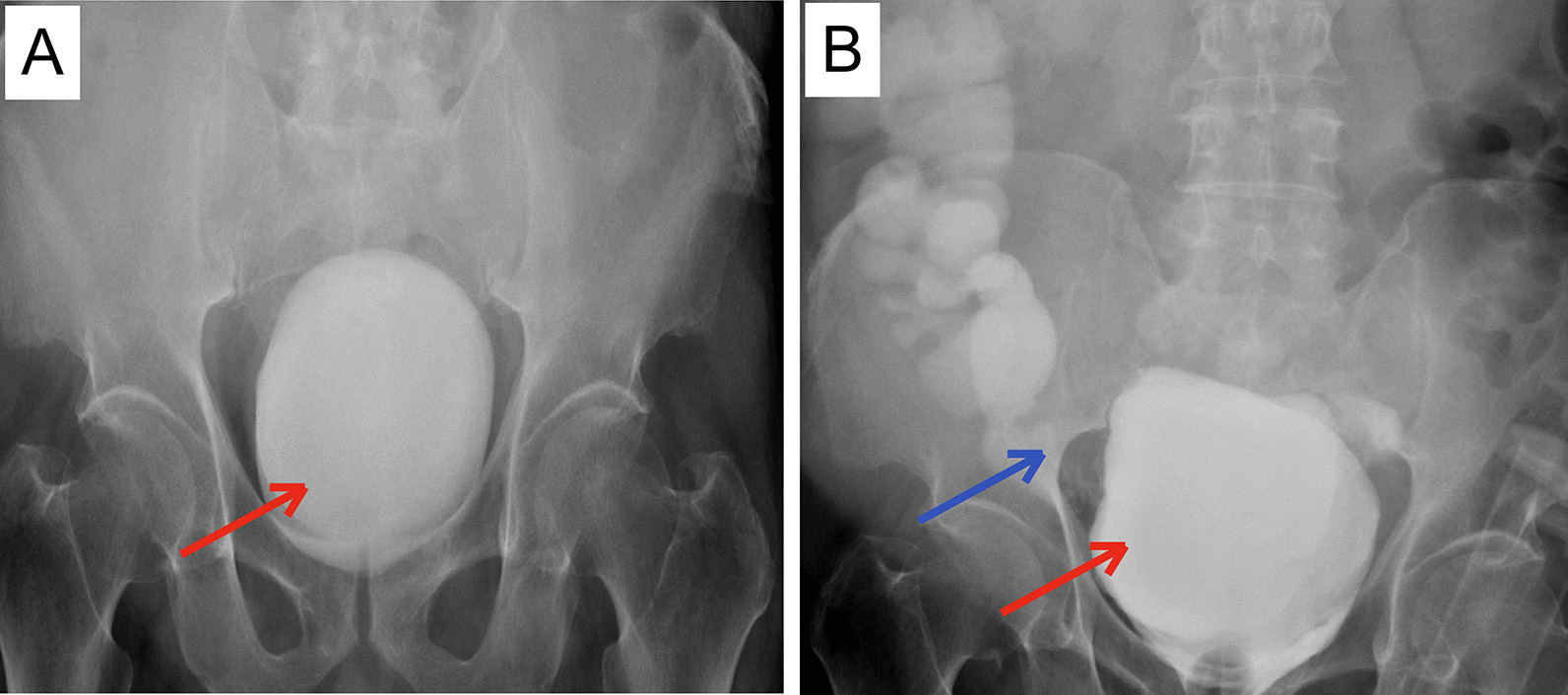
Pelvic X-rays before (**A**) and after (**B**) meglumine angiography. Notes: The red arrows in the X-film **A** and **B** both indicate giant stones. After injecting a meglumine contrast agent, the contrast material entered the adjacent small intestine and colon, indicated by the blue arrow

The patient's urine output was low due to severe diarrhoea, so routine urine tests could not be performed. A urine culture was positive for *Escherichia coli*. A faecal culture was negative for both *Escherichia coli* and *Vibrio cholerae.* A pelvic CT showed a large single stone in the bladder, with a small amount of gas in the cavity; it showed thickening of the bladder wall and inflammatory changes (Fig. 1). No obvious fistula was found in the colon or rectum by electronic colonoscopy. The contrast medium flowed into the ileum through the neovesical ileum via an indwelling 14F catheter for retrograde cystography (Fig. 2).

The patient was diagnosed with a "bladder stone, ileal neobladder fistula, and fistula located in the ileum". The patient underwent "fistulectomy, cystotomy, partial ileectomy, and end-to-end ileal anastomosis”. The abdominal longitudinal incision was opened, and the ileum and ileal neobladder were clearly adhered to the abdominal wall. A calculus was seen, its size was approximately 9.0 × 9.0 cm, and the surface was smooth; the bladder calculi were completely removed, and the fistula at the bottom of the neobladder was found to be connected with the end of the ileum. The oral segment was surgically severed and excised, and the severed end was subjected to ileal end-to-end anastomosis. The operation was successful, and the patient had no symptoms of diarrhoea after 3 months of follow-up. Colour Doppler ultrasound showed that the shape of the neobladder was intact, and no rupture was found 3 months after the operation.

## Discussion and conclusions

Radical cystectomy (RC) and urinary diversion (UD) are the main treatment methods for muscle invasive bladder cancer. The surgical steps are complicated and usually take a long time. Postoperative complications such as small bowel obstruction or leakage, urinary leakage or fistula occur at a rate of approximately 3% [5]. To reduce the occurrence of postoperative intestinal complications, ureterocutaneostomy seems to be a feasible option in frail elderly patients [6]. Compared to open techniques, robot-assisted UDs are able to improve intraoperative blood loss and postoperative recovery [7], and in more than 60 surgical cases, reduced the high level of postoperative complications [8]. Urinary calculi are delayed complications of orthotopic neobladder construction after total cystectomy. Bladder stones are a rare complication of ileal neovesical fistula, which can cause neovesical cutaneous fistula [4].

INF mainly manifests as recurrent urinary infections, dysuria, watery stools, abdominalgia and other symptoms [9, 10]. It is difficult to diagnose through routine examination and is easily misdiagnosed as acute gastroenteritis. This patient suffered from chronic diarrhoea, which was misdiagnosed as “acute gastroenteritis or gastrointestinal dysfunction” in the early stage, and the symptoms were not relieved after treatment with empirical antibiotics and intestinal flora regulation. Finally, the diagnosis was made only after neocystography showed “vesical ileostomy”. Diarrhoea caused by INF is considered to be related to the fact that urine enters the intestine through the fistula and stimulates the intestinal mucosa. Part of the cause of INF is necrosis of the intestinal mucosa after radiotherapy. In this case, the bladder stone may compress the neobladder mucosa, resulting in ischaemic necrosis of the local tissue, followed by a fistula.

The ileal neobladder is adjacent to the colon and rectum[11], so these patients are also easily misdiagnosed as having vesicocolic fistula. The diagnosis of INF mainly depends on gastroenterography, neocystography and colonoscopy [12]. Because the fistula is located in the ileum, colonoscopy has difficulty finding the fistula. Gastroenterography and neocystography are helpful to determine the location of the fistula. In this case, the fistula position was finally found by neocystography.

Surgery is an effective treatment for INF. The treatment process of INF is easy for colovesical fistula, which can be performed by one-stage ileal end-to-end anastomosis without temporary enterostomy [13]. The key to the operation is to excise the fistula and double-layer suture the neobladder and ileum. In addition to the above surgical plan, this patient required a neobladder incision and lithotomy. During the 3-month follow-up after surgery, the patient no longer had any gastrointestinal symptoms, such as diarrhoea.

INF is a rare complication after RC. For patients with a history of RC operation, INF needs to be considered for symptoms such as recurrent urinary infections and diarrhoea. Surgery is the main treatment for INF and can achieve a good prognosis.

## Data Availability

Data sharing was not applicable to this article, as no datasets were generated or analysed during the current study.

## Abbreviations

RC: Radical cystectomy
INF: Ileal neobladder fistula
